# Supplementary material for: TGFβ1, MMPs and cytokines profiles in ocular surface: Possible tear biomarkers for pseudoexfoliation
Source: PLoS One. 2021 Apr 29;16(4):e0249759. doi: 10.1371/journal.pone.0249759 (PMC8084233; doi:10.1371/journal.pone.0249759)
Supplement: S1 Raw images — Zymographic gel image of (a) tears; (b) aqueous humor; (c) serum; (d & e) blot of FN and Gapdh; lane 1, control; lane 2, PXF; lane 3, PXG. (PDF) [file pone.0249759.s001.pdf]

S1 Raw images:

a, Zymographic gel image of tears

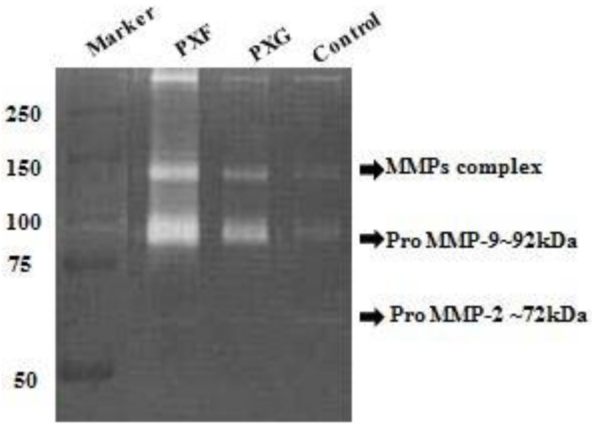

b, Zymographic gel image of aqueous humor

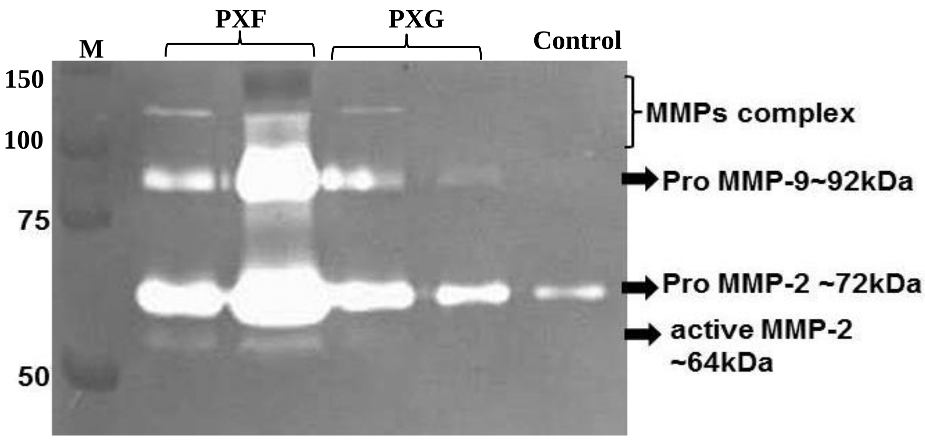

c, Zymographic gel image of serum

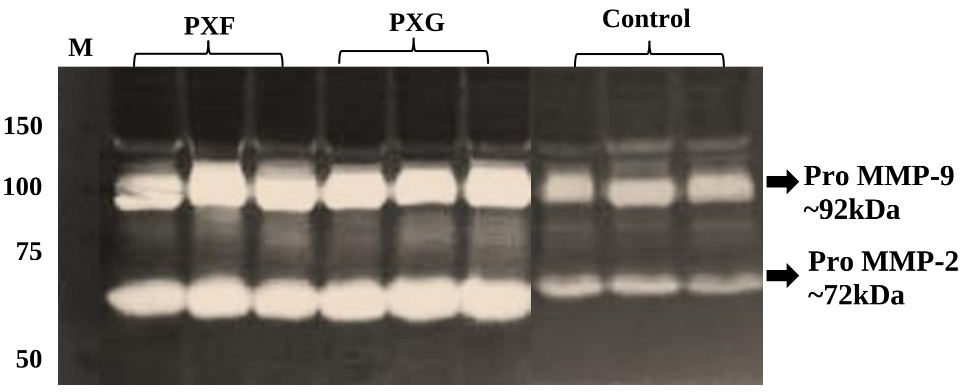

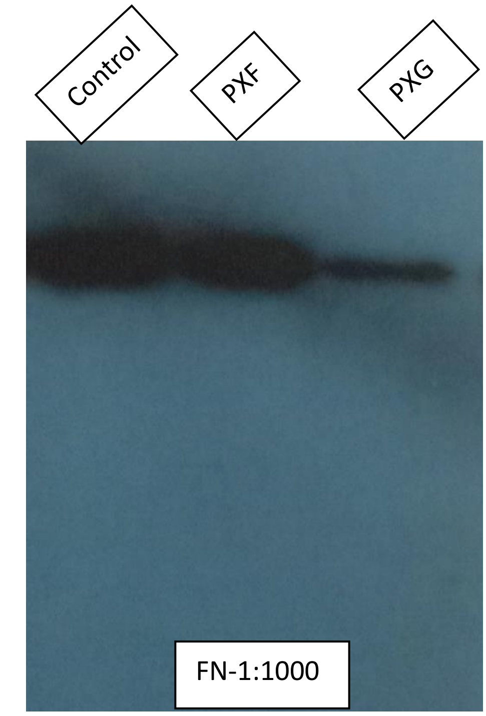
Control
PXF
PXG
FN-1:1000

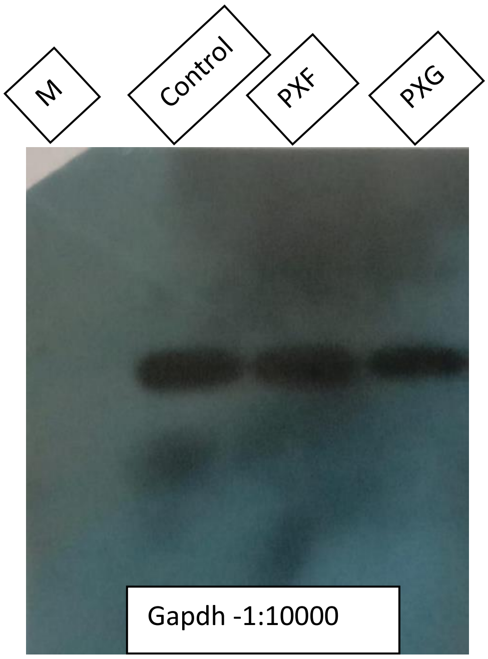
M
Control
PXF
PXG
Gapdh -1:10000
