# Supplementary material for: TGFβ1, MMPs and cytokines profiles in ocular surface: Possible tear biomarkers for pseudoexfoliation
Source: PLoS One. 2021 Apr 29;16(4):e0249759. doi: 10.1371/journal.pone.0249759 (PMC8084233; doi:10.1371/journal.pone.0249759)
Supplement: Attachment — Submitted filename: Response to Reviewer.docx [file pone.0249759.s003.docx]

**Author's Response To Reviewer Comments:**

**Journal Requirements:**

1. Please ensure that your manuscript meets PLOS ONE's style requirements, including those for file naming.

Author response: We have checked the journal requirement and my manuscript meets PLOS ONE’s style requirements.

2. PLOS ONE now requires that authors provide the original uncropped and unadjusted images underlying all blot or gel results reported in a submission’s figures or Supporting Information files.

Author response: We have already added original uncropped and unadjusted blot images in supplemental information as S1 Figure in my original manuscript and in the revised manuscript we have added uncropped and unadjusted gel image as S2 Figure (legend was added in the end of manuscript where Supplemental information is given) present in the supplemental information. Further we ensure that my figures in the result section adhere fully to journal guidelines and we provided the original underlying images for all blot or gel data reported in my submission.

3. We noticed you have some minor occurrence of overlapping text with the following previous publication(s), which needs to be addressed:

Author response: In our revise manuscript I cited all other sources (including your own works), and rephrased the duplicated texts in the beginning of result (line no 262-267) and discussion (line no 385-391) section which is highlighted.

**Reviewers' comments:**

**Review Comments to the Author**

Reviewer #1: The manuscript reports changes in tear profile in Pseudoexfoliation for onset of glaucoma. It is suggested that authors revise English and avoid using complex sentences in addition to clarifying some of the points listed below:

Author response: We have professionally edited the manuscript for grammar and used simple sentences as suggested.

Lines 51-52 - Instead of asking questions please define these topics for the reader at the outset.

Author response: The sentences have been rephrased and defined the disease for better understanding.

Line 56 - increased in what?

Author response: “Vascular phenomenon such as vasospasm, systemic hypertension, angiographic vascular perfusion defects and oxidative stress markers such as generation of reactive oxygen species (ROS) have been found to be increased which partly explain the high incidence of ischemic ocular and systemic events associated with endothelial dysfunction seen characteristically in PXF”. The sentence is changed as per your suggestion in the revised manuscript.

Line 57 - which disease? Try to write in simple clear sentences.

Author response: The sentence is changed as per your suggestion in the revised manuscript.

Lines 57-58 - 'Though this disease' which disease?

Author response: We are mentioning about PXF as disease in the sentence. The sentence is changed as per your suggestion in the revised manuscript.

Line 59 - which entity?

Author response: We are addressing PXF here as “this entity”. We have changed the sentence accordingly as per your suggestion in the revised manuscript.

Line 62 - what does 'This' refers to?

Author response: 'This' refers to PXF. We have added PXF in the sentence and rephrased it accordingly as per your suggestion.

Line 62-67 - Complex sentence. Please write simple sentences.

Author response: We have completely rephrased the sentences in the revised manuscript.

Line 69 - 'would be probably hold differences' - not clear

Author response: We want to say that there would be different proteins or markers expressed in different tissues of different stages of PXF disease that would be interesting to know.

Line 78 - what or which?

Author response: Typo error. We have changed in the revised manuscript.

Lines 103-104 - Write in simple sentences - The aim of the current study was...

Author response: We have changed the sentence accordingly as per your suggestion in the revised manuscript.

Line 105 - 'In brief' - this is rather explanatory than brief

Author response: We have changed the sentence accordingly as per your suggestion in the revised manuscript.

Line 120 - please specify if the diagnosis was done in this study

Author response: We have included “the diagnosis was done in this study” and changed the sentence accordingly as per your suggestion in the revised manuscript.

Line 153 - procedure for what was followed?

Author response: Procedure of the isolation of protein from the schirmer strip was followed. We have included about the procedure in the revised manuscript.

Line 170 - which guideline? Writing name of the manufacture in brackets will be good.

Author response: We have mentioned about the guidelines described in R&D System. R&D system is the manufacturer of the ELISA kit.

Line 201 - 'examined examination' - correct the sentence

Author response: We have changed the sentence accordingly as per your suggestion in the revised manuscript.

Table 1 - Any reasoning for high male vs female numbers?

Author response: Since most of our patients of pseudoexfoliations are farmers by occupation, it is logical to expect more of male than females.

Line 312 - 'surprisingly' - state what was the expectation otherwise?

Author response: We wanted to say that the previous result of serum is consistent with the tears and aqueous humor of TGFβ1 ELISA results. There is a typo error we have changed the “surprising” word with “as expected” in the revise manuscript.

Line 402 - 'Earlier studies' - mention the reference

Author response: I have added reference in the revised manuscript.

Line 436 - 'release of iris pigment release' - correct the sentence

Author response: We have changed the sentence accordingly as per your suggestion in the revised manuscript.
